# Metallic Nanostructures Based on DNA Nanoshapes

**DOI:** 10.3390/nano6080146

**Published:** 2016-08-10

**Authors:** Boxuan Shen, Kosti Tapio, Veikko Linko, Mauri A. Kostiainen, Jari Jussi Toppari

**Affiliations:** 1 Nanoscience Center, Department of Physics, University of Jyväskylä, P.O. Box 35, Jyväskylä 40014, Finland; kosti.t.o.tapio@jyu.fi; 2 Biohybrid Materials, Department of Biotechnology and Chemical Technology, Aalto University, P.O. Box 16100, Aalto 00076, Finland; veikko.linko@aalto.fi (V.L.); mauri.kostiainen@aalto.fi (M.A.K.)

**Keywords:** DNA nanotechnology, DNA origami, self-assembly, metallization, nanoelectronics, plasmonics, nanoparticle

## Abstract

Metallic nanostructures have inspired extensive research over several decades, particularly within the field of nanoelectronics and increasingly in plasmonics. Due to the limitations of conventional lithography methods, the development of bottom-up fabricated metallic nanostructures has become more and more in demand. The remarkable development of DNA-based nanostructures has provided many successful methods and realizations for these needs, such as chemical DNA metallization via seeding or ionization, as well as DNA-guided lithography and casting of metallic nanoparticles by DNA molds. These methods offer high resolution, versatility and throughput and could enable the fabrication of arbitrarily-shaped structures with a 10-nm feature size, thus bringing novel applications into view. In this review, we cover the evolution of DNA-based metallic nanostructures, starting from the metallized double-stranded DNA for electronics and progress to sophisticated plasmonic structures based on DNA origami objects.

## 1. Introduction

Microelectronics have become an inseparable part of our lives by providing increasingly powerful and portable energy-efficient devices. This development has been enabled and driven by constant scaling down of the components and by building integrated circuits (IC) from these pieces. During the early stages, the semiconductor industry adopted an observation that later has turned into a principle, which is generally known as Moore’s law [1,2]: the number of transistors in the ICs roughly doubles every two years. To sustain this trend, the further scaling down of the components of the ICs, including insulating oxide layers, semiconducting channels and, especially, metallic interconnections, is essential and therefore of great interest.

On the other hand, metallic nanostructures are well known for their fascinating optical properties, which are attributed to the excitation of localized surface plasmon resonances (LSPR) [3,4]. They have promising applications in the enhancement of optical signals in fluorescence [5,6,7], Raman [8,9,10] and IR spectroscopy [11,12], as well as sensing based on the change in the refractive index [13,14]. Surface plasmons can be understood as collective oscillations of free electrons in metal, and therefore, their properties are highly dependent on the size, shape and material of the nanoparticles. Thus, the field of plasmonics would benefit greatly from the capability to fabricate high-resolution metal nanostructures with arbitrary shapes in a parallel fashion.

Further development of the abovementioned branches demands scaling down of the metal structures. However, during recent years, the evolution of microfabrication techniques has decelerated due to the fundamental limitations of the traditional top-down lithography methods. For example, UV lithography can achieve relatively high resolution, but requires tremendously expensive instruments, whereas raster methods (e-beam, focused ion beam, etc.) are limited by pixel size and are usually remarkably slow as a result of the serial processing. Although these techniques are developed constantly, the necessity for alternative bottom-up-based methods is increasingly growing. In order to genuinely fabricate ICs using nanoscale components, new emerging methods are urgently needed.

An intriguing approach to tackle these issues would be to exploit self-assembly and molecular-scale structures in creating novel nanomaterials. One promising research field that provides highly parallel, accurate and programmable fabrication of nanoscale objects is the structural DNA nanotechnology, which has enjoyed remarkable progress during the last decade [15,16]. In the structural DNA nanotechnology, DNA molecules are used as a construction material rather than mere carriers of the genetic information. Due to their superior programmability, DNA molecules can form arbitrary 2D and 3D objects with sub-nanometer-level precision in a parallel manner. However, DNA is not metallic. Although the electrical conductivity of DNA molecules and structures still remains a bit of a controversial topic, it seems that the DNA-based nanostructures have rather low conductivity [17,18,19,20]. This makes the usage of DNA structures in nanoelectronics limited, but not at all impossible [21]. Conceivable solutions would be to convert precise DNA nanoshapes into metallic ones while retaining the structural details or to transfer the shape of the origami to metallic nanostructures.

In this review, we first discuss the current state of the metallization of DNA nanostructures and focus especially on methods, which could preferably produce continuous metallic nanostructures. In the following section, we give an overview about structural DNA nanotechnology, including the basics of how DNA nanostructures can be conjugated with metallic nanoparticles (Section 2). Section 3 covers the chemical metallization methods for simple DNA molecules, as well as for more advanced DNA nanostructures. Furthermore, in Section 4, a novel DNA mold-casting method is discussed. Finally, in Section 5, a technique that enables the fabrication of metallic nanoscale patterns on substrates by combining the DNA nanotechnology with traditional microfabrication processes is presented.

## 2. Structural DNA Nanotechnology

### 2.1. DNA Self-Assembled Nanostructures

The concept of using DNA as a structural material was first conceived of by Nadrian Seeman about 30 years ago [22]. He proposed that single-stranded DNA (ssDNA) molecules with partially complementary sequences could form a branched structure via Watson–Crick base pairing. Further, these branched structures could self-assemble into 2D or 3D lattice structures. Since then, a completely new research field, structural DNA nanotechnology, has emerged, and it has lately enjoyed an accelerated progress. At the early stages, relatively rigid designs of double-crossover (DX) and triple-crossover (TX) tiles were predominantly used. These programmable tiles can assemble into 2D nanoribbons, nanotubes [23,24] and into 3D crystalline structures [25]. The field has undergone a second blooming phase since the invention of large-scale non-periodic DNA structures, which resemble the traditional Japanese art called origami [26]. In the original design of the DNA origami, a long viral ssDNA (scaffold) is folded into a desired 2D shape with the hybridization of dozens of unique oligonucleotides (staples). Followed by the 2D shapes, the technique has been generalized to 3D shapes and curved structures [27,28,29]. Later on, the method of using oligonucleotides as 2D and 3D bricks has enabled scaffold-free fabrication of nano-objects with diverse shapes by simply selecting the desired strands (pixels or voxels) from the molecular canvas (full set of bricks) [30,31]. Very recently, top-down methods for creating meshed DNA origamis have been presented [32,33]. In these techniques, computer algorithms are used to route the scaffold and the staple strands, and therefore, they allow the fully-automated design of complex DNA nanoshapes [33].

The power of the DNA nanotechnology lies not only in the highly versatile design motifs, but also in the addressability and modularity of the created structures. Each unique oligonucleotide in a DNA nano-object can be modified with various functional groups, allowing the structure to be functionalized by, e.g., metallic nanoparticles (NPs) [34,35], enzymes [36,37,38,39] and carbon nanotubes [40,41]. Especially in the case of metallic NPs, the formed assemblies can be directly used as plasmonic devices or they can be utilized as seeds for the growth of continuous metal structures, as discussed below. In the next subsection, we will briefly discuss the basics of the conjugation of DNA nanoshapes with metallic NPs.

### 2.2. Conjugation of DNA Nanostructures with Metallic NPs

The utilization of chemically-prepared metal nanoparticles (MNP) has gained wide interest over recent decades [42,43]. Typically, these particles are formed by first reducing metal salts to zerovalent metal atoms, followed by a reduction or growth step, where metal ions collide and form larger clusters called nuclei seeds. These seeds continue to grow as long as there are excess metal ions. Furthermore, by selecting suitable surfactants or reagents, one can control the growth directions of the nuclei seeds. To date, various MNPs with diverse shapes have already been produced, including, e.g., spherical particles [44,45,46], triangles [47], rods [48], hexagons [49] and rectangles [50]. For practical use, it is necessary to stabilize the nanoparticles using protective agents or layers. There are two types of stabilization methods: electrostatic and steric stabilization. In the former one, an ionic double layer is created to induce repulsion and to shield particles from each other. In the latter case, particles are coated with organic molecules to prevent agglomeration. In addition, one can combine the surface stabilizing agents to produce directional growth, as mentioned above.

Functionalization of MNPs is typically achieved through a chemical modification of the surface of the nanoparticles, for example by substituting the protective or capping layer of the nanoparticle with ligand molecules that have the desired properties. This was first introduced by Brust et al. [51]. Usually, the functionalization process consists of several steps: chemisorption of the molecule to the surface of the MNP followed by straightening reorientation of the molecule. The chemisorption is generally a fast reaction that lasts only a few minutes [42], but the following steps can take several hours or even days. Ligands are usually attached via a terminal or linker group. One example of such a linker group is a thiol, which forms a covalent bond to gold. Thiols are extremely feasible, since gold is a widely-used material in MNPs, and the sulfur-gold bond is found to be one of the strongest in nature [52]. This means that the thiol can relatively easily substitute any ligand or ions on the surface of the gold nanoparticles (AuNP). Other possible linkers include, e.g., disulfides [53], phosphine [54] and amines [55].

In catalysis research and surface engineering, most commonly-employed nanoparticles are indeed AuNPs owing to their straightforward and robust fabrication and versatile modification possibilities [45]. In particular, AuNPs functionalized with thiolated oligonucleotides are extensively used in DNA nanotechnology, since they can be easily attached to a DNA strand and furthermore linked to the DNA structure via sequence-complementarity. In addition, there exists a wide variety of other functionalized nanoparticles, such as thiol-PEG-coated spherical gold nanoparticles [56] and Au rods [57], alkylamine-stabilized platinum nanoparticles [58], benzyl methacrylate (BzMA) hollow Au@SiO_2_ particles [59], oligo-functionalized Au-triangles [60], silica-coated silver nanoparticles [61] and AgNPs/graphene composition functionalized with streptavidin [62], that can be utilized in a wide range of applications, for example in energy storage materials and catalysis [63,64].

## 3. Chemical Metallization of DNA Nanostructures

### 3.1. Metallization of dsDNA and ssDNA

Early research on the metallization of DNA focused on a direct chemical metallization of double-stranded DNA (dsDNA) molecules. The usual method can be divided into three steps: (1) initial binding of seed ions or complexes onto DNA; (2) the subsequent reduction of the seeds into nucleation clusters; and (3) the growth of these nucleation clusters into a metallic structure by reduction reactions. The seeding of DNA can be executed via implantation of either plain metal ions or metal complexes onto the negatively-charged DNA backbone by metal ion bonding or by linking the seeds to DNA using a suitable reaction. After seeding, more metal is reduced to the seeds. Typically, this reduction step is chemically driven, but in some metallization schemes, UV light can be used for the reduction. The seeding and the following reduction can be carried out in the solution phase or on the substrate. All of the following metallization protocols of dsDNA use silicon dioxide as a substrate if not otherwise stated.

**Figure 1:**
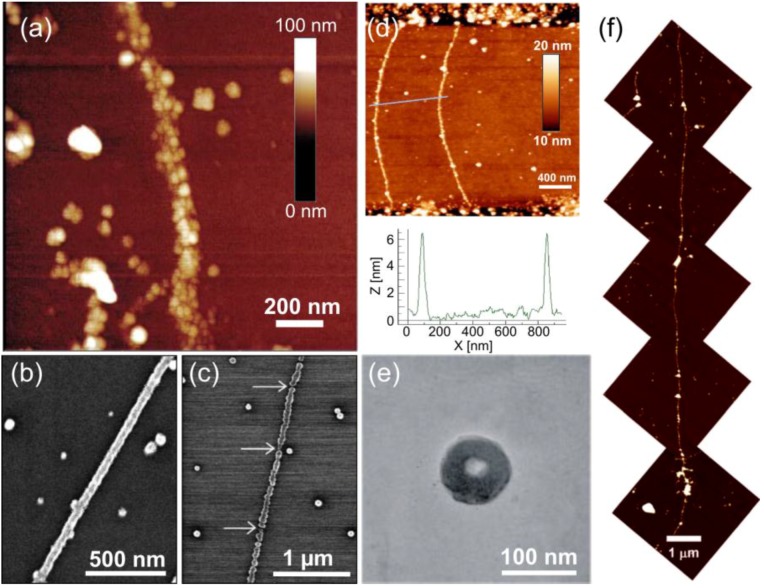
(**a**) Atomic force microscope (AFM) image of silver nanowires [65]; (**b**) continuous Pd nanowires using a 42-h incubation time and a 45 °C temperature [69]; (**c**) when lowering the incubation time to 20 h, but using the same temperature, discontinuous Pd nanowires were formed [69]; (**d**) continuous Pt nanowires formed by UV photoexcitation [83]; (**e**) silver toroids formed by reducing silver salt on spermine treated ssDNA [77]; (**f**) long Ni nanowires on lambda-DNA [72]. (a) is reproduced with permission from [65]. Copyright Nature Publishing Group, 1998; (b,c) are reproduced with permission from [69]. Copyright John Wiley and Sons, 1998; (d) is reproduced with permission from [83]. Copyright Elsevier, 2009; (e) is reproduced with permission from [77]. Copyright American Chemical Society, 2005; (f) is reproduced with permission from [72]. Copyright American Chemical Society, 2006.

The first DNA metallization was realized by Braun et al. [65], who demonstrated the fabrication of silver nanowires on λ-DNA (a linear dsDNA from lambda phage) scaffolds. The seeding was achieved by direct binding of Ag^+^-ions to the DNA backbone via electrostatic interaction, and hydroquinone was subsequently used to reduce the ions to form continuous, grainy silver wires (see Figure 1a). Reduction of both the bound silver ions and loose silver ions in the vicinity of the backbone resulted in wires with a width of ~100 nm and an average grain size of 30–50 nm (the produced wire in Figure 1a is at least 12 μm in length). Puchkova et al. [66] continued along these lines by investigating the impact of the substrate on the reduction reaction. They found that a negatively-charged silicon oxide surface itself could act as a reducing agent. Wirges et al. [67] introduced an alternative pathway to form linear, pearl necklace-shaped nanowires, by binding two- and four-ion silver clusters onto DNA. This protocol utilized the well-known reaction of monoaldehyde and dialdehyde molecules and the Tollens solution, where the silver clusters act as nucleation centers for the following growth process. Since then, a plethora of different linear dsDNA-based metal nanowires has been presented. It should be mentioned here that usually the attachment scheme of the initial nucleation sites differs from metal to metal.

Metallization of palladium on DNA has been reported by Richter et al. [68] and Nguyen et al. [69], whereas Mertig et al. [70,71] have used platinum in DNA metallization. Palladium and platinum are chemically similar, since both have a complex form in the seeding process, and typically, the same reduction agent (dimethylaminoborane (DMAB)) can be employed to form a metallic wire. Similarly as before, λ-DNA was used as a scaffold for direct binding of Pt- and Pd-complexes: the DNA was incubated in a palladium chloride or platinum chloride solution, followed by the reduction into a metallic Pt or Pd wire in the presence of DMAB. Nguyen et al. studied the effect of the seeding time and temperature on the integrity of the nanowire (see Figure 1b,c). They discovered that longer seeding times (~42 h) and slightly elevated temperature (45 °C) produced well-formed, continuous wires (Figure 1b), whereas shorter times and either too high or low temperatures resulted in grainy and discontinuous wires (Figure 1c). The wires in Figure 1b had a mean width of 50 nm and a maximum length of 3 μm, whereas the grainy wires as in Figure 1c had a mean width of 35–40 nm and a maximum length of 1 μm. The conductivity measurements of the wires in Figure 1b revealed resistances in the range of kΩ, which further emphasizes the quality of these nanowires. Mertig et al., for one, described the pathway of the platinum-DNA binding kinetics: the PtCl_2_(H_2_O)_2_ complexes bind first to the N7 position of guanine, followed by N7 of adenine and then to all other positions of all of the bases.

Becerril et al. [72] have demonstrated that DNA can be equally used to fabricate nickel wires. Again, λ-DNA was employed to form linear Ni-nanowires. The substrate with immobilized DNA was incubated in a nickel chloride or nitride solution, and subsequently, the Ni^2+^ ions were reduced into metal using sodium borohydride. The procedure typically resulted in a long nickel wire with a length of over 10 μm and a height of about 12 nm, as illustrated in Figure 1f.

Other studies include nanowires made of zinc oxide by Atanasova et al. [73], gold nanowires and networks by Swami et al. [74] and Fischler et al. [75], as well as copper nanowires by Monson et al. [76]. Swami et al. used aurichloric acid to bind gold ions to herring testes DNA and reduced the bound ions into gold nanowires and networks using sodium borohydride.

One challenge in the metallization of DNA is to avoid residual metal cluster formation on the substrate (see Figure 1a–d). Swami et al. utilized tetraoctylammonium bromide (TOAB) to extract excess gold clusters and ions in the solution phase, thus producing more pure nanowires, usually 5–10 nm in width. In contrast to this, Fischer et al. produced 300 nm-long and 8 nm-high gold nanowires using the Tollens reaction to form first silver clusters and further reducing gold ions onto them. Studies by Monson et al. [76] involved λ-DNA incubation in copper(II) nitride solution and a succeeding reduction using ascorbic acid. The reduction was performed in a step-like fashion. After the first copper reduction, the wires had an average height of 3.03 nm, and after the second reduction, the height was slightly increased to 3.15 nm. Copper has lower affinity towards DNA than other metals, and hence, a couple of seeding steps was required to get even half of the DNA strands metallized.

More recently, studies on the direct metallization of ssDNA have been conducted. Approaches by Zinchenko et al. [77], Chen et al. [78] and Pu et al. [79] are based on the condensation of unfolded ssDNA (T4 DNA) into a toroidal shape using a tetravalent cation spermine (SPM), followed by the abovementioned metallization methods. For example, Pu et al. were able to produce gold toroids by incubating T4 DNA in aurichloric acid solution and by reducing gold ions using UV-light (254 nm). Zinchenko et al. created silver toroids by seeding and using sodium borohydride-based reduction (see Figure 1e). The gold toroid fabricated by Pu et al. had a 90-nm outer diameter and a 30-nm inner diameter with a thickness of 30 nm, while Zinchenko et al. reported an average outer diameter of 93 ± 7 nm and an average inner diameter of 22 ± 4 nm.

An alternative route to utilize ssDNA in the metallization and functionalization was introduced by Keren et al. [80], when they used a nucleoprotein filament (ssDNA polymerized with RecA) as a site-specific mask in a dsDNA metallization. The filament will bind to the section of the dsDNA scaffold with the complementary or nearly complementary sequence to the filament, replacing the segment of the other strand of the scaffold. This creates the section along the dsDNA, where there are three DNA strand stacked together. This assembly can subsequently be used either directly to form nanowires with a gap in the filament position or as a platform to conjugate AuNPs site-specifically to the dsDNA parts. In the case of nanowires with a gap, an aldehyde-derived λ-dsDNA (48,502 bases) was used as a scaffold to attach Ag ions. When the 2027 nucleotide-long filament is bound to the dsDNA, the RecA will disable the reduction of the Ag ions, thus leaving a void of Ag seeds in the dsDNA. The Ag seeded dsDNA, except the section bound with filament, was further grown larger by electroless gold deposition, which resulted in a 50-nm-high gold-wire with an insulating gap.

Besides the chemical reduction methods, UV light can be equally employed in DNA metallization as briefly mentioned above. Berti et al. [81], Yang et al. [82] and Erler et al. [83] have reported the fabrication of platinum and silver nanowires by UV photoexcitation. Seeding was carried out similarly as mentioned above, i.e., by using platinum chloride, platinum nitride or silver nitride. However, samples were irradiated by 254 nm UV light, because DNA can act as a photosensitizer owing to its UV absorption around 260–280 nm [84]. The process is described by a two-photon reaction, where metal ions are reduced in the presence of the electrons generated by photo-oxidation of the DNA bases [84,85,86]. This treatment typically yields similar grainy nanowires as in the case of chemical reduction. Erler et al. reported the fabrication of Pt-nanowires with the height of 10 nm using λ-DNA across electrode gaps (see Figure 1d), whereas Yang et al. produced a series of Pt-networks or clusters. Berti et al., for one, created narrow, only 1.5–3 nm-thick silver nanowires using λ-DNA as a template. Overall, UV-photoreduction offers a possible pathway to form nanostructures without introducing any extra chemicals.

### 3.2. Tile-Based DNA Nanostructures

Since the chemical metallization methods of dsDNA and ssDNA are merely based on the interaction between metal-ions and DNA molecules, it is straightforward to extend these techniques to more complex DNA nanostructures. Well before the DNA origami technique was invented, attempts to metallize tile-based DNA nanostructures were presented. Usually, the tile-based assemblies do not have a defined size due to their periodicity, in other words they either form 2D network structures or lattices, 1D nanoribbons or nanotubes. In this subsection, some results of metallizing such structures are discussed.

**Figure 2:**
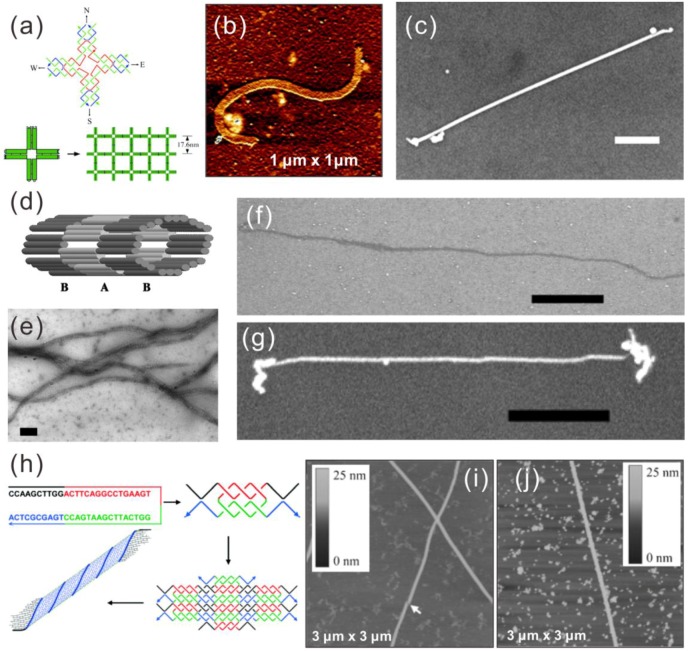
Upper panel (**a**–**c**) [23]: (a) schematic of a 4 × 4 tile and nanoribbon assembly form from these tiles; (b) AFM image of a nanoribbon; (c) SEM image of a metallized silver nanoribbon, scale bar 500 nm; middle panel (**d**–**g**) [87]: (**d**) scheme of a nanotube made of TX tiles; (e,f) TEM and SEM image of nanotubes; (g) SEM image of a metallized silver nanotube; scale bars in (e–g) are 100 nm, 1 μm and 1 μm, respectively; lower panel (**h**–**j**) [24]: (h) assembly model of a nanotube from a single oligonucleotide with palindromic sequence, (i) AFM image of the nanotube; (**j**) metallized Pd nanotube. (a–c) are reproduced with permission from [23]. Copyright The American Association for the Advancement of Science, 2003; (d–g) are reproduced with permission from [87]. Copyright National Academy of Sciences, USA, 2004; (h–j) are reproduced with permission from [24]. Copyright John Wiley and Sons, 2006.

As early as 2003, LaBean et al. successfully metallized a DNA nanoribbon structure with silver using a modified “two-step metallization” [23] in which the silver seeding was done in an aqueous solution instead of on a substrate. In their work, the nanoribbons comprised of 4 × 4 DNA tiles were metallized into continuous wires with a height of 35 ± 2 nm, a width of 43 ± 2 nm and lengths up to ~5 μm, as shown in Figure 2a–c. In addition, the electrical conductivity of some of the wires was characterized, yielding ohmic behavior with corresponding bulk resistivity of 2.4 × 10^−6^ Ω∙m. They reported high reproducibility of the nanowires and much higher conductivity than the silver-metallized dsDNA nanowires.

Later in 2004, LaBean et al. metallized DNA nanotubes self-assembled from thiol-modified triple-crossover (TX) tiles, which contained three co-planar double helices linked together at four crossover points [87]. They used the same silver two-step method as previously and acquired continuous nanowires with a height of ~35 nm, a width of ~40 nm and a length of up to ~5 μm, as depicted in Figure 2d–g. However, two terminal current-voltage (I-V) measurements with electrodes fabricated by electron beam lithography (EBL) showed resistivity one order higher than the abovementioned nanowires made from the nanoribbons.

In 2006, Mao et al. designed a double-crossover (DX) tile-like structure comprised of just a single oligonucleotide [24]. The strand consists of four palindromic segments, thus making it complementary to itself (Figure 2h). A two-strand complex with two duplex domains and four single-stranded overhangs can form at native conditions. These complexes will further assemble into a 2D lattice via the hybridization of overhangs and eventually form a tubular and more stable structure. AFM imaging has shown tubes up to 60 μm in length and around 6 nm in height with varied widths from 30–70 nm (Figure 2i). Metallization of these DNA nanotubes was carried out on a mica substrate. Immobilized nanotubes were incubated in a Pd^2+^-ion solution followed by a chemical reduction to form metallic nanowires. The Pd nanowires produced by this method were 30–80 nm wide and up to 30 μm long, as seen in Figure 2j. They have heights between 10 and 18 nm, and SEM imaging showed that the wires were composed of Pd grains of 30–60 nm in diameter compactly deposited along the DNA tube. The electrical conductivity of such wires remains unknown, since electrical measurements were not performed.

Although these works only presented wires with indefinite lengths, the tile-based nanowires served as a major step further towards the fabrication of metallic structures with all three dimensions truly at the nanoscale. Many of these methods can be generalized to metallization of structurally more complex assemblies, such as DNA origami nanostructures, as discussed in the next section. Nevertheless, the issues with granularity caused by the randomized nucleation sites in the chemical metallization have been partially solved. To form continuous metal nanostructures, several metal reduction steps or overgrowing of nanowires are needed. However, these methods result in blobby extrusions of the samples, limiting the resolution of the obtained metallic nanostructures.

All of the aforementioned approaches (ssDNA, dsDNA and tile-based) are mainly dealing with linear structures with only a little possibility to control the shape and the size of the pattern, like in the case of toroids by Zinchenko et al. [77], Chen et al. [78] and Pu et al. [79]. These kinds of structures are well suited for applications in nanoelectronics, and they can be used as transistors or nanowires, but less so for plasmonic applications, where the shape and the size of the structure have a strong influence on the optical properties of the structure. One possible way to utilize DNA in plasmonics is based on the DNA origami technique, which will be the topic of the following sections.

### 3.3. DNA Origami Metallization

Since the invention in 2006, DNA origami has been extensively used owing to its programmability and addressability properties. The desire to transform these well-defined sub-100 nm DNA origami structures into metallic shapes has produced numerous DNA origami metallization procedures. However, there have been certain challenges in the metallization, namely the stability of the origami during the metallization process or the poor adhesion of DNA origami to a surface. Moreover, the increased selectivity requirements due to its miniaturized size may cause problems.

**Figure 3:**
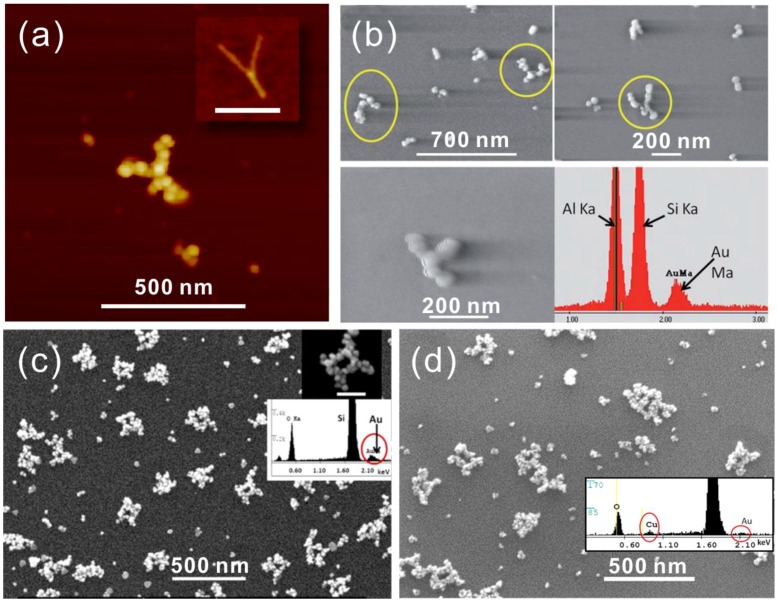
(**a**) AFM image of a Y-shaped DNA origami metallized with Au on mica. The origami shape before metallization is presented in the inset (scale bar 200 nm) [88]. (**b**) DNA origami seeded with Pd^2+^ and metallized with Au and the corresponding EDX results [89]. (**c**,**d**) A circuit-like DNA origami metallized with Au (c) and Cu (d) with respective EDX as the inset [90]. (a) is reproduced with permission from [88]. Copyright American Chemical Society, 2011; (b) is reproduced with permission from [89]. Copyright Royal Society of Chemistry, 2011; (c,d) are reproduced with permission from [90]. Copyright American Chemical Society, 2013.

In 2011, Woolley et al. reported the first successful metallization of a Y-shaped DNA origami using a two-step method (Ag seeding, Au growth) on a substrate with little or no background metallization, as shown in Figure 3a [88]. To increase the stability of origamis during the process, they used Mg^2+^ containing buffer for rinsing and dialysis steps; the added Mg^2+^ is essential in preventing origami unfolding due to the unscreened repulsive force. Moreover, they studied the influence of the absolute concentration of staple strands on the stability during the dialysis process. In addition, the adhesion of origami to the substrate was sustained by adding MgCl_2_ to the electrodeless Au plating solution. To further improve the selectivity, they used more concentrated DNA origami solution with 10:1 staple to scaffold ratio, a smaller volume and longer rinsing time. Although some of the seeded origamis were removed, they indeed achieved relatively high selectivity.

Later in the same year, Woolley et al. published another Pd-based method for rapid DNA origami metallization [89]. In this paper, they not only reduced the process time, but also managed to increase the metallized particle density on the surface, as shown in Figure 3b. This method consists of three steps: (1) Pd activation; (2) Pd reduction; and (3) electrodeless plating [68,69]. The authors found that the activation times beyond 10 min did not significantly increase the seeding density, but too long an activation time could cause DNA to be partially removed from the substrate. Therefore, it was concluded that 10–30 min was the sufficient activation time, and thus, the duration of the process could be greatly shortened. In this work, the important role of Mg^2+^-ions in the procedure was also reported. A 10 mM concentration of Mg^2+^ in Au plating solution allows Pd-seeded DNA to remain on the surface without affecting the plating effectiveness. Here, an interesting discovery was that the nucleation site density was higher on a DNA origami than on a λ-DNA under identical seeding conditions, which implies that denser origami structures, e.g., 3D DNA origami, may provide smoother structures and even better results.

In 2013, the same group demonstrated successful Au and Cu metallization of Pd seeded circuit-like DNA origami [90]. It is noteworthy that this was the first demonstration of electrically conductive Cu nanostructures fabricated on a DNA origami template. After the initial Pd seeding, they employed several additional seeding steps to produce less grainy structures. In their Au plating experiments, they compared a procedure that is based on a readily available kit to a process where a commercial solution and another Au plating method was used [91,92]. Both resulted in continuous metal structures, but the latter one showed a larger grain size, as shown in Figure 3c,d. The authors also reported that an enlargement of the attached Pd seeds with a short Au plating was needed for a better Cu deposition. Both the Au and Cu plated origamis were electrically characterized using nanoelectrodes fabricated by EBL. The average resistivity of Au and Cu structures were 11 × 10^−5^ Ω∙m and 3.6 × 10^−4^ Ω∙m, respectively.

### 3.4. Metallization Based on Nucleation on Functionalized Nanoparticles

One compelling route to incorporate the nucleation seeds into the DNA origami scaffold is to utilize the selective DNA base pairing. For DNA origamis, it is straightforward to extend staple strands in such a way that they appear complementary to the strands attached to functionalized nanoparticles. Therefore, the nanoparticles can be positioned along the origami scaffold with extremely high precision.

**Figure 4:**
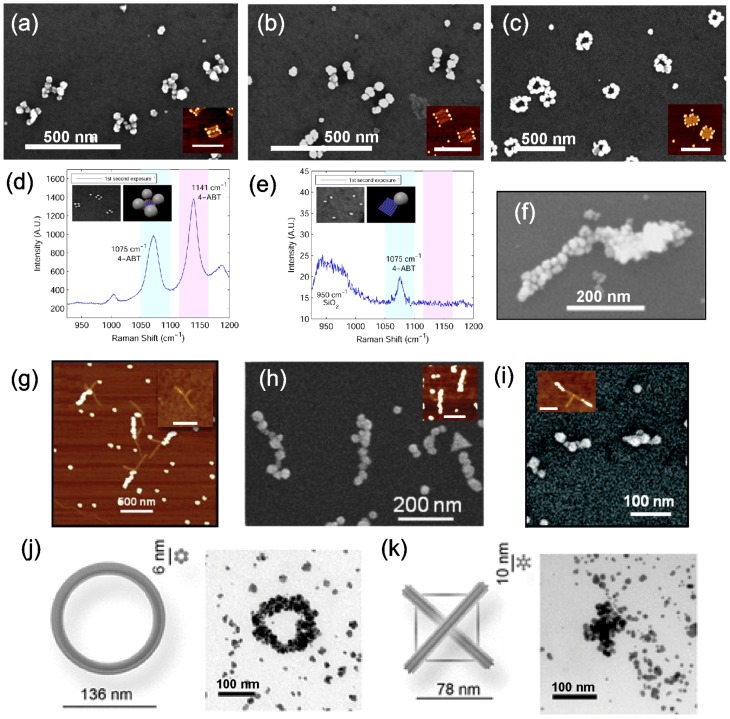
(**a**–**c**) Metallized H-shapes, two parallel bars and rings. Insets are the corresponding structures before metallization, scale bars are 250 nm [93]. (**d**) SERS spectrum of aminobenzenethiol obtained by using the four-corner bound AuNPs structure (inset) for enhancement of the Raman signal. (**e**) Structures with just a single AuNP resulted in an insignificant SERS signal [94]. (**f**) A single origami bar metallized with both gold and copper on either side [96]. (**g**–**i**) T-shaped DNA structures with AuNPs bound to one branch, both branches (inset in (h)) and only on the edges of the T-branches (inset in (i)). The structures in the insets of (h) and (i) were further metallized into continuous metal structures as shown in (h) and (i). Scale bars in insets of (g) and (h) are 500 nm and of (i) 100 nm [91]. (**j**,**k**) Ring and cross patterns fabricated by reducing gold seeds that were bound via electrostatic attraction [97]. (a–c) are reproduced with permission from [93]. Copyright American Chemical Society, 2011; (d–e) are reproduced with permission from [94]. Copyright American Chemical Society, 2014; (f) is reproduced with permission from [96]. Copyright American Chemical Society, 2014; (g–i) are reproduced with permission from [91]. Copyright American Chemical Society, 2012; (j,k) are reproduced with permission from [97]. Copyright John Wiley and Sons, 2011.

Indeed, Pilo-Pais et al. [93] have fabricated different patterns of AuNPs on a rectangular origami including H-shapes, two parallel bars, four-corner bound AuNPs structures and ring-like structures, as shown in Figure 4a–c. Here, the staple strands at the desired AuNP binding sites were modified to have two 29-nucleotide (nt) long extensions. The extensions consisted of a TTTTT spacer followed by a 24 base-long sequence complementary to the oligonucleotides conjugated with AuNPs. Attachment of AuNPs via hybridization and the subsequent silver metallization was performed on both mica and SiO_2_ substrates. SEM images show that after the metallization, the structures still retain their distinct features, although the results show granular nanostructures, where the seed particles were 50 nm. Furthermore, four-corner bound AuNP origami structures (see Figure 4d) were utilized in the calibration of the growth speed of the AuNPs, where a roughly linear dependence on time versus the size of the nanoparticles was discovered.

Later, Pilo-Pais et al. [94] returned to this matter by utilizing the four-corner bound AuNP origami in a study of detecting aminobenzenethiol (4-ABT) by surface-enhanced Raman spectroscopy (SERS) (see Figure 4d). In SERS, the hot spots, i.e., localized strong plasmon fields near nanostructures and especially the so-called gap modes between them, are used to enhance the Raman scattering of the studied molecules. Here, each nanoparticle bound to a corner of the rectangular origami creates gap mode hot spots with its nearest neighbors, which induces about a hundred-times larger signal enhancement per particle compared to a single particle case (see Figure 4e). These results demonstrate that origamis can be exploited in the formation of efficient SERS probes. Further, by using origami assembly, the metallic nanoparticle composition and its shape can be matched to the chosen molecule and scattering scheme to produce an immense SERS signal.

Pearson et al. [91] demonstrated a similar binding scheme on a T-shaped DNA origami, where either an individual branch, both branches or just the edges of the T-branches were separately conjugated with functionalized AuNPs (see Figure 4g and the insets in Figure 4h,i). Sticky ends were placed along the long edge of the T origami, so that the spacing between them was 11 nm. After the AuNP conjugation to the origami, metallization was carried out using a commercial kit combined with the plating protocol by Natan et al. [95] with the varied duration of the treatment. Again, nanostructures formed with shorter treatment were discontinuous, while longer treatment times yielded grainy, but continuous nanostructures, as shown in Figure 4h,i. The average width of the wire in Figure 4h was 33 nm with the standard deviation of 7.3 nm. The continuity of the sample was confirmed by measuring the I-V-characteristics revealing kΩ-range resistance, similar to EBL-fabricated nanowires.

Harb et al. pushed the boundaries of this technique even further in 2014, when they developed a method to specifically metallize the same origami structure with two different metals [96]. The key aspect of this work was the use of octadecanethiol in between the site-specific Au plating and unspecific Cu plating. AuNPs functionalized with complementary DNA (cDNA) sequences were first hybridized to one-half of a bar-like origami followed by an Au plating with a commercial kit. Then, the octadecanethiol was added to the sample surface to cover the gold structure from further seeding and metallization. Finally, the ionic Pd seeding and Cu plating were performed on the unprotected side of the origami resulting in a Au-Cu junction, as demonstrated in Figure 4f. The SEM images showed two distinct morphologies and contrast to prove a successful plating. The work possesses potential applications for example in the fabrication of nanoscale thermocouples.

In addition to the complementary DNA scheme, one can utilize functionalization based on the charge of nanoparticles. In other words, one can take advantage of an electrostatic attraction between the negatively-charged DNA and the particles. Liedl et al. [97] demonstrated that amine-coated, positively-charged, tiny gold clusters can be seeded into the negatively-charged DNA backbone. Distinct DNA origami patterns, e.g., ring and cross patterns (see Figure 4j,k), were used as scaffolds. Gold clusters bound along the origami were chemically reduced into continuous metal structures using the commercial Nanoprobe kit. The authors reported that the fabricated structures retained their original features, as long as the substructure size was larger than 50 nm.

The success of the electrodeless metallization of the DNA origami has made it possible to fabricate arbitrarily-shaped metallic structures under the 100 nanometer scale by the bottom-up. However, the granular appearance of the structures is still one of the intrinsic problems of the electrodeless plating method. Still, when compared to the initial research on ssDNA, dsDNA and tile-based DNA-structures that face the same problem of granular appearance, DNA origami offers a more versatile toolset to fabricate defined size structures for both electronics and plasmonic applications, e.g., for SERS, as demonstrated by Pilo-Pais et al. [94], or for the fabrication of double metal junctions by Harb et al. [96].

## 4. Casting of Nanoparticles Using DNA Molds

Besides using DNA origamis as templates for creating metal nanostructures, origamis can be used as molds to confine the growth of metallic nanoparticles. In this method, a AuNP seed is attached inside a hollow 3D DNA origami cavity. By using a chemical metal ion reduction, the single metallic nanoparticle grows inside the origami chamber to the shape prescribed by the mold. By this approach, the resulting nanoparticles are homogeneous, since they circumvent the abovementioned problem of having multiple nucleation sites all over the DNA origami. Nevertheless, the resolution of this method is still limited by the stiffness of the DNA origami as a mold, and particles with very sharp corners or ridges are fairly challenging to fabricate by this method.

**Figure 5:**
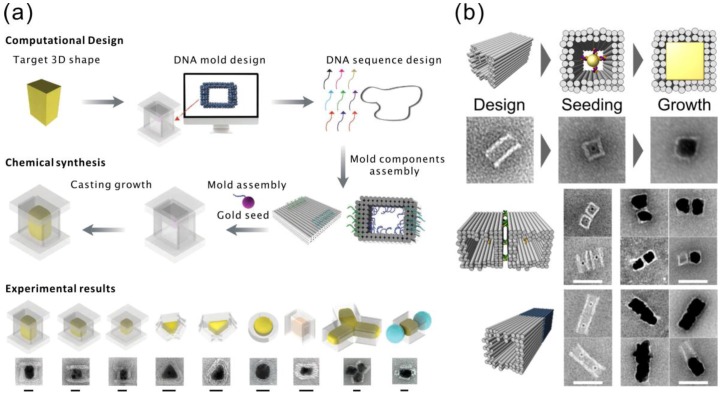
(**a**) Top panel: casting metal particles with prescribed 3D shapes using programmable DNA nano-structure molds. Bottom panel: experimental results of the cast procedure. The scale bars are 20 nm [98]. (**b**) Top panel: schematic views and TEM images of the AuNP seed grown inside the DNA origami mold without the lid. Bottom panel: side-by-side and head-to-tail designs. All scale bars correspond to 50 nm [99]. (a) is reproduced with permission from [98]. Copyright The American Association for the Advancement of Science, 2014; (b) is reproduced with permission from [99]. Copyright American Chemical Society, 2014.

Two groups of researchers published almost at the same time their research on this subject. In detail, Yin et al. [98] designed barrel-like structures with DNA handles pointing into the cavities for hybridization of the cDNA functionalized AuNPs. After the conjugated AuNPs were attached inside the origami cavities, DNA origami lids were mixed with the solution. The barrel openings at the ends of the origami were covered by these lids, which resulted in a sealed chamber with typically one AuNP seed inside. The seed was then grown with AgNO_3_ using ascorbic acid as a reducing agent. The modularity of this method was demonstrated by fabricating three Ag nano-cuboids with different aspect ratios and other shapes, including triangles, discs and complexes of different particles, as shown in Figure 5a. In addition to the TEM micrographs, the electromagnetic behavior and the plasmonic spectra further proved the feasibility of the proposed technique. The aforementioned silver structures have a maximum yield of roughly 40% according to the authors. Moreover, not only single particles, but also composites of NPs can be fabricated by this method. The authors have shown several designs with more than one cavity segment linked together, yielding more complex NP structures. Interestingly, the authors reported that it was particularly difficult to grow gold nanoparticles with this method, possibly due to the chelating effect caused by ethylenediaminetetraacetic acid (EDTA) in the buffer of the gold precursors. Removing EDTA could improve the growth rate of the gold nanoparticles; however, the yield is still significantly lower than for the silver structures (only 6%).

On the other hand, Seidel et al. successfully fabricated cuboid gold nanostructures with a similar method, but a different reductant (hydroxylamine). In their work, tube-like DNA origamis with quadratic cross-sections were used as molds [99]. AuNP seeds were attached via cDNA strands, similarly as demonstrated by Yin et al. The reducing agent hydroxylamine was premixed with the AuNP attached origamis, and HAuCl_4_ was gradually added after that. By doing so, the growth tends to self-terminate due to the limited amount of gold ions in solution. The authors observed enlarged particles and somewhat thinner origami walls in some of the samples, which indicate that a single layer of DNA origami cannot completely stop the growth of gold. Therefore, better control over the shape of the nanoparticles may require multiple layers of DNA origami as a mold. In addition, the overgrown cuboid particles tend to connect to each other along the open cavity axis, which may be due to the lack of a capping agent. Seidel et al. further tested the composition of such mold-casted nanoparticles by designing a side-by-side pair and a head-to-tail pair, yielding promising results (see Figure 5b).

Despite the challenges raised by the limited stiffness of DNA origami molds, these methods enable nanoparticle synthesis with homogenous composition and 3D features. Moreover, the 3D origami not only works as a mold, but the staples can also serve as anchors for further functionalization, which enables the assembly of multiple metallic nanoparticles and even nanoparticles containing different materials. Therefore, mold-casting can yield implementations for both nanoelectronics and plasmonics, owing to the variety of possible devices that can be directly assembled with this method.

## 5. DNA Nanolithography

To eliminate the granularity and other issues in the electrodeless metallization via reduction, completely different approaches have been developed. Besides the mold-casting method described above, a DNA nanolithography that combines the DNA self-assembly with conventional lithography, is another alternative route to make nanoscale patterns on substrates. In this approach, DNA or DNA nanostructures are used as a mask or their pattern is converted to a mask. Physical vapor deposition (PVD) is then used to deposit metal through these masks to form the nanoscale patterns. PVD methods, like sputtering and evaporation, are already quite advanced techniques, which have been an essential part of the integrated circuit industry for decades. For example, metal films produced by PVD are usually very smooth and continuous, unlike the chemically-grown ones. Moreover, the thickness of these films can be controlled with a sub-nanometer precision. Besides a deposition mask, the hard mask converted from the DNA nanostructures can also be used as an etching mask. Herein, we review some of the results falling into the category of DNA nanolithography.

### 5.1. dsDNA and DNA Nanogrid as Masks in Lithography

**Figure 6:**
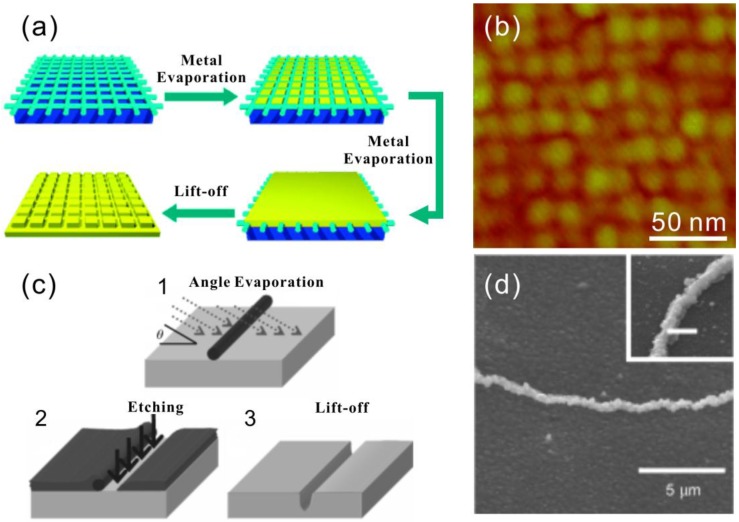
(**a**) Method to transfer the negative pattern from the DNA nano-grid to a gold surface [100]; (**b**) AFM image of the gold surface with the negative square grid from DNA assembly [100]; (**c**) by using a dsDNA as a mask in an angled evaporation, an open area is formed on the evaporated film, which can be further utilized as an etching mask [101]; (**d**) silver nanowire grown in the etched trench [101]. (a,b) are reproduced with permission from [100]. Copyright John Wiley and Sons, 2004; (c,d) are reproduced with permission from [101]. Copyright John Wiley and Sons, 2007.

Mao et al. demonstrated a novel route to replicate the pattern of DNA self-assembled structures as a negative image on metal film [100]. They successfully replicated a DX tile array, a 1D DNA triangle array, a tetragonal 2D DNA and pseudohexagonal 2D arrays on a gold substrate. To make these patterns, a continuous gold film was thermally evaporated onto a mica surface with predeposited DNA structures. Afterwards, the metal film was peeled off by stripping the solidified epoxy between the sample and a glass slide. On the backside of the film, gold had dents on the places where the DNA was originally located. The fabrication steps are depicted in Figure 6a. By this method, 1D or 2D DNA nanostructures (Figure 6b) can be replicated having roughly the original dimensions.

Woolley et al. employed λ-DNA on silicon substrate as a mask in an angled metal evaporation. The idea is that silicon substrate in the shadow of the DNA molecule does not get covered with metal during the evaporation (see Figure 6c) [101]. This exposed part of the substrate can be subsequently etched by reactive ion etching (RIE) into nanotrenches with linewidths as narrow as 7 nm. The trenches can be subsequently used as templates for a silver electroless plating (Figure 6d) or they can be closed by a thin layer of oxide to form nanochannels.

The usage of DNA molecules or DNA structures as direct masks is indeed quite an innovative approach; however, it is unfortunately restricted by the fact that the DNA molecule is chemically instable in many common microfabrication processes, let alone that they are very likely to detach from the substrate in solution-based processes. In addition, 2D DNA structures have a very limited height (~2 nm), which makes it inadequate as a mask for many applications.

### 5.2. Silica Mask from DNA Origami for Metal Evaporation

The limitation of bare DNA as a mask has motivated researches to transfer the DNA patterns to materials like silicon oxide, which are more durable and widely used in microfabrication processes. Taking advantage of the difference in water affinity between a DNA molecule and the substrate material, either etching or growth can be used to selectively make SiO_2_ masks with highly accurate patterns inherited from the DNA nanostructures, especially in the case of DNA origami. In this subsection, a couple of mask fabrication recipes and an example of how customized metallic nanoshapes can be fabricated by these masks are discussed.

**Figure 7:**
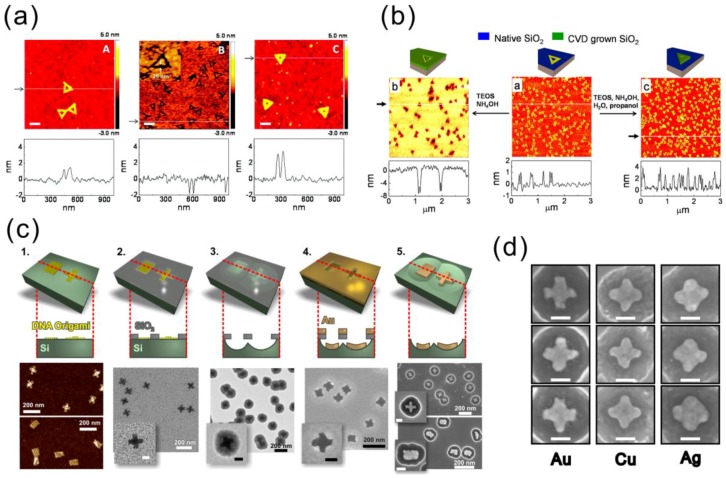
(**a**) DNA origami-modulated etching of SiO_2_ by HF vapor [102]; (**b**) room-temperature CVD process for SiO_2_ growth using DNA origami as a mask [104]; (**c**) fabrication steps to produce high-resolution metallic shapes on the Si surface using DNA origami stencils [105]; (**d**) similar cross-shaped structures fabricated from different metals via the same origami mask [105]. (a) is reproduced with permission from [102]. Copyright American Chemical Society, 2011; (b) is reproduced with permission from [104]. Copyright American Chemical Society, 2013; (c,d) are reproduced with permission from [105]. Copyright Royal Society of Chemistry, 2015.

Surwade et al. used DNA origami to modulate the etching rate of SiO_2_ when etched by HF vapor [102]. As a result, the shape of a triangular DNA origami was very precisely transferred into the SiO_2_ as either a negative or a positive tone pattern, as seen in Figure 7a. The vapor-phase etching of SiO_2_ using HF gas needs water as a catalyst. Compared to the DNA molecule, the amount of adsorbed water on SiO_2_ is lower at high humidity and higher at low humidity levels. Thus, the local etching rate of SiO_2_ between the DNA covered area and exposed area is different. Therefore, either trenches or ridges can be produced. Under optimized conditions [103], triangular trenches with an 11.8 ± 0.3 nm depth can be obtained after 20 min of etching with a selectivity of 2.73. It has been noticed that even an individual DNA scaffold loop can be seen in the transferred pattern. Furthermore, the SiO_2_ mask was used in the RIE process to etch the silicon substrate with a depth of 8.4 ± 2.7 nm.

Besides the etching modulation, Surwade et al. found that the DNA origami could also affect the growth rate of SiO_2_ and TiO_2_ at room temperature in a tetraethyl orthosilicate (TEOS)-based chemical vapor deposition (CVD) process, as demonstrated in Figure 7b [104]. A variety of substrates, including Si wafer, mica and gold, can be used in this CVD process. The basic chemical reaction to grow SiO_2_ by TEOS involves water as a reactant and NH_3_ as a catalyst. The CVD can be accomplished using an easily accessible setup, in which vials of TEOS and NH_4_OH are placed in the glass desiccator together with the sample containing the deposited DNA origamis. A negative-tone growth can take place without any further treatments, whereas positive-tone growth requires elevated humidity and propanol vapor. Moreover, positive-tone TiO_2_ patterns were successfully created on a silicon wafer with titanium isopropoxide (Ti(OiPr)_4_) as the precursor. The introduction of this room temperature CVD method to generate the inorganic oxide mask with nanometer-precise custom-shaped patterns has opened the door to utilize DNA self-assembly in the conventional microfabrication industry.

Shen et al. took one step further and demonstrated the feasibility of the aforementioned SiO_2_ masks in the fabrication of metal nanostructures (gold, copper and silver) [105] on a silicon substrate, as shown in Figure 7c,d. Two different 2D DNA origami shapes, rectangular and cross-shaped, were utilized. In this work, the authors introduced cured silica gel as a humidity buffer to improve the reproducibility of the SiO_2_ mask. After the mask formation, the silicon substrate underneath the oxide was isotropically etched using RIE, which yielded a hemispherical cavity under each origami pattern. Metal was subsequently deposited through the mask openings by e-beam evaporation followed by the removal of the SiO_2_ mask by either HF or HF/HCl wet etching. Metallic nanostructures with sub-20-nm features were successfully fabricated inside silicon bowls with high yields (~90%). In principle, any metallic nanoparticle that can survive the HF wet etching can be fabricated by this method. Extending the fabrication protocol on a flat transparent substrate would enable optical measurements and numerous applications, such as SERS and fluorescence enhancement of the molecules.

This seemingly eccentric method, which combines the DNA self-assembly and conventional microfabrication processes, may readily find applications in the field of nano-optics and plasmonics. This parallel manufacturing method could be combined with large-scale cost-effective deposition techniques [106], and therefore, it could yield presumable industrial innovations.

### 5.3. Patterning of Graphene with Metallized DNA Nanostructures

Based on the same idea, i.e., to produce a more durable mask from DNA nanostructures, Jin et al. demonstrated that chemically-metallized DNA origami and single-strand tiles (SSTs) can be used as a positive etching mask to pattern graphene on a substrate [107]. In this work, the authors first treated monolayer graphene films on SiO_2_/Si substrate with 1-pyrenemethylamine methanol solution to improve affinity with the DNA structures. Then, they attached glutaraldehyde-treated DNA origamis (O-shaped) or SSTs with letter shapes (X, Y, L, etc.) to the graphene surface. The following metallization of the DNA nanostructures was carried out on-site via a two-step method using silver as seeds and a commercial kit for gold growth (as in Section 3.3). The metallized gold nanostructures followed the shape of the original DNA nanostructures and served as masks in a subsequent O_2_ RIE process that removes the unprotected parts of the graphene. Finally, the gold masks were dissolved with a 0.1 M NaCN solution, and only the graphene patterns under them were left on the surface. Jin et al. have used a convolution model to describe the spatial information transfer in each lithography step and pointed out that the metallization distorted the information the most due to the granularity and enlargement of the pattern. Raman spectra after each fabrication step were also shown to prove the reduced dimensions of graphene.

By transferring the high-resolution spatial information from DNA nanostructures to graphene, Jin et al. created a link between the highly programmable DNA nanostructures to the promising 2D conductive material, which could benefit both fields. In addition, similar methods could be used to pattern other 2D materials, such as MoS_2_ and BN, for large-scale high-resolution electronic devices fabrication.

In the DNA nanolithography, masks made from DNA nanostructures, especially the silica masks, provide much less distortion in the pattern transfer, which enables the fabrication of semi-2D metallic nanostructures with precise size and shape. Therefore, especially plasmonic applications, due to their strong dependence in the structure geometry, can benefit greatly from such methods. However, unlike the mold-casting method, after converting the DNA nanostructures into hard masks, they will lose their addressability and modularity, which inhibits further site-specific functionalization.

## 6. Conclusions

The capability to precisely control the dimensions, shape and position of functional groups has made DNA nanostructures exceptionally powerful in nanotechnology. Nevertheless, to reach the maximum potential of these nanostructures in nanoelectronics and plasmonics, strategies to incorporate different metals into them or fully metallize the structures have been developed. By conjugation of MNPs with DNA nano-objects, plasmonic nano-devices can be fabricated, but for nanoelectronics, more continuous structures are needed. The direct route to achieve this is electroless chemical metal plating on seeds. However, the seed-based metallization schemes suffer from granular structures and discontinuities, which is highly undesired especially for applications where the conductivity of the wires or networks is essential. On the other hand, it could be an advantage for obtaining high SERS signals. Controlled growth of MNPs by the 3D DNA origami mold serves as a highly ingenious approach to form arbitrarily-shaped metallic nanoparticles. Combined with the programmability of the DNA origami, numerous possibilities are foreseen in the future. Finally, by using a hard stencil mask with DNA origami-shaped openings, metallic structures with sub-20-nm resolution can be fabricated on the surface via regular PVD methods. Albeit that the fabrication method is limited to substrates, the smoothness and homogeneous composition of the produced nanostructures make the method have a high potential for both nanoelectronics and plasmonics. Along these lines, we expect that all of these conceivable techniques to fabricate metallic nanostructures by means of DNA self-assembly will be significantly developed in the near future. Therefore, we strongly believe that these structures will ultimately find a plethora of uses in the field of nanoelectronics and plasmonics.

## Abbreviations

The following abbreviations are used in this manuscript:

λ-DNA	a linear dsDNA genome from a bacterial virus called lambda phage
2D	two-dimensional
3D	three-dimensional
AFM	atomic force microscopy
AuNP	gold nanoparticle
B-DNA	double-helical DNA in the B-form (geometry attribute)
BzMA	benzyl methacrylate
cDNA	complementary DNA
CVD	chemical vapor deposition
DNA	deoxyribonucleic acid
DMAB	dimethylaminoborane
dsDNA	double-stranded DNA
DX	double-crossover
EBL	electron beam lithography
EDTA	ethylenediaminetetraacetic acid
EDX	energy-dispersive X-ray spectroscopy
I-V	current-voltage
MNP	metal nanoparticle
NP	nanoparticle
nt	nucleotide
PEG	polyethylene glycol
PVD	physical vapor deposition
RIE	reactive ion etching
SEM	scanning electron microscopy
SPM	spermine
SERS	surface-enhanced Raman spectroscopy
ssDNA	single-stranded DNA
TEM	transmission electron microscopy
TEOS	tetraethyl orthosilicate
TOAB	tetraoctylammonium bromide
TX	triple-crossover
UV	ultraviolet
